# Phase Segregation in PdCu Alloy Nanoparticles During CO Oxidation Reaction at Atmospheric Pressure

**DOI:** 10.1002/advs.202302663

**Published:** 2023-06-28

**Authors:** Yingying Jiang, Alvin M. H. Lim, Hongwei Yan, Hua Chun Zeng, Utkur Mirsaidov

**Affiliations:** ^1^ Department of Physics National University of Singapore Singapore 117551 Singapore; ^2^ Centre for BioImaging Sciences Department of Biological Sciences National University of Singapore Singapore 117557 Singapore; ^3^ Department of Chemical and Biomolecular Engineering College of Design and Engineering National University of Singapore Singapore 119260 Singapore; ^4^ Centre for Advanced 2D Materials and Graphene Research Centre National University of Singapore Singapore 117546 Singapore; ^5^ Department of Materials Science and Engineering National University of Singapore Singapore 117575 Singapore

**Keywords:** bimetallic nanoparticle, CO oxidation, operando TEM, segregation

## Abstract

Bimetallic nanoparticle (NP) catalysts are widely used in many heterogeneous gas‐based reactions because they often outperform their monometallic counterparts. During these reactions, NPs often undergo structural changes, which impact their catalytic activity. Despite the important role of the structure in the catalytic activity, many aspects of how a reactive gaseous environment affects the structure of bimetallic nanocatalysts are still lacking. Here, using gas‐cell transmission electron microscopy (TEM), it is shown that during a CO oxidation reaction over PdCu alloy NPs, the selective oxidation of Cu causes the segregation of Cu and transforms the NPs into Pd–CuO NPs. The segregated NPs are very stable and have high activity for the conversion of CO into CO_2_. Based on the observations, the segregation of Cu from Cu‐based alloys during a redox reaction is likely to be general and may have a positive impact on the catalytic activity. Hence, it is believed that similar insights based on direct observation of the reactions under relevant reactive conditions are critical both for understanding and designing high‐performance catalysts.

## Graphical abstract

This study with operando transmission electron microscopy approach shows that during a CO oxidation reaction over PdCu alloy nanoparticles, selective oxidation and segregation of Cu transform the nanoparticles into very stable and active Pd–CuO nanocatalysts for CO to CO_2_ conversion reaction.

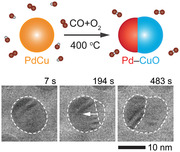

## 1 Introduction

Bimetallic alloy nanoparticles (NPs) are attractive candidates for a wide range of heterogeneous catalytic reactions, such as CO oxidation,^[^ ^1^, ^2^ ^]^ steam reforming,^[^ ^3^, ^4^ ^]^ and water–gas shift reactions,^[^ ^5^, ^6^ ^]^ due to their high activities,^[^ ^1^, ^2^ ^]^ tunable selectivities,^[^ ^5^ ^]^ and low costs.^[^ ^1^, ^2^ ^]^ During reactions under gaseous environments and at elevated temperatures, the structure of alloy NPs is susceptible to change, which can often boost their catalytic activity^[^ ^1^, ^7^, ^8^ ^]^ and selectivity.^[^ ^9^ ^]^ These changes in structure and reactivity of the nanocatalysts are due to segregation,^[^ ^9^, ^10^ ^]^ oxidation,^[^ ^1^, ^4^, ^8^ ^]^ or reduction^[^ ^7^ ^]^ of their metallic components. Thus a comprehensive understanding of the structural changes of the NPs under catalytic environments and how such changes impact their activities is critical to designing and optimizing the alloy NP catalysts for industrial applications.^[^ ^11^, ^12^ ^]^

Recent experimental studies using environmental transmission electron microscopy (TEM),^[^ ^13^, ^14^ ^]^ ambient pressure X‐ray photoelectron spectroscopy (XPS),^[^ ^1^ ^]^ and scanning tunneling microscopy (STM)^[^ ^15^ ^]^ revealed the structural changes of alloy nanocatalysts during CO oxidation reaction. These in situ studies showed that any change, such as the formation of an oxide layer on the surface of the alloy NPs (i.e., CoO*_x_* on PdCo,^[^ ^1^ ^]^ NiO*_x_* on PtNi,^[^ ^15^ ^]^ and CuO*_x_* on AuCu^[^ ^13^, ^16^, ^17^ ^]^) is caused by the adsorption of O_2_ to the NP surface. However, the approaches used in these studies have certain limitations. First, a direct correlation between the NP structures and their real‐time activities is missing in these studies.^[^ ^1^, ^13^, ^15^, ^16^, ^17^, ^18^ ^]^ Second, the gas pressures utilized in these studies are very low (<1 Torr)^[^ ^1^, ^13^, ^14^, ^15^ ^]^ in comparison to the relevant gas pressures used in industrial catalytic reactions, which are typically in the range of 5–100 bar,^[^ ^19^ ^]^ and this three‐orders‐of‐magnitude pressure difference (< 1 Torr vs a few bars) can result in completely different catalyst structures during the reactions.^[^ ^20^ ^]^

Because of these limitations, two key questions regarding the alloy NPs in catalytic reactions remain unanswered. First, how does the reactive gas environment modify the structures of the individual NPs? Second, what is the effect of these structural changes on the catalytic activities? In order to address these questions, it is imperative to monitor individual NP catalysts under relevant reaction conditions. Here, using gas‐cell TEM combined with an inline mass spectrometer,^[^ ^9^, ^21^, ^22^, ^23^, ^24^, ^25^, ^26^ ^]^ which enables to study nanomaterials in a gaseous environment that closely mimics conditions of real catalytic reactions,^[^ ^19^ ^]^ we tracked the evolution of PdCu and RhCu NPs during a CO oxidation reaction (i.e., 2CO + O_2_ → 2CO_2_)^[^ ^27^ ^]^ at atmospheric pressures. We chose PdCu and RhCu NPs as the catalysts because, among a variety of alloy NPs, they are the best catalysts for CO oxidation reaction.^[^ ^2^, ^28^ ^]^

## 2 Results and Discussion

**Figure 1:**
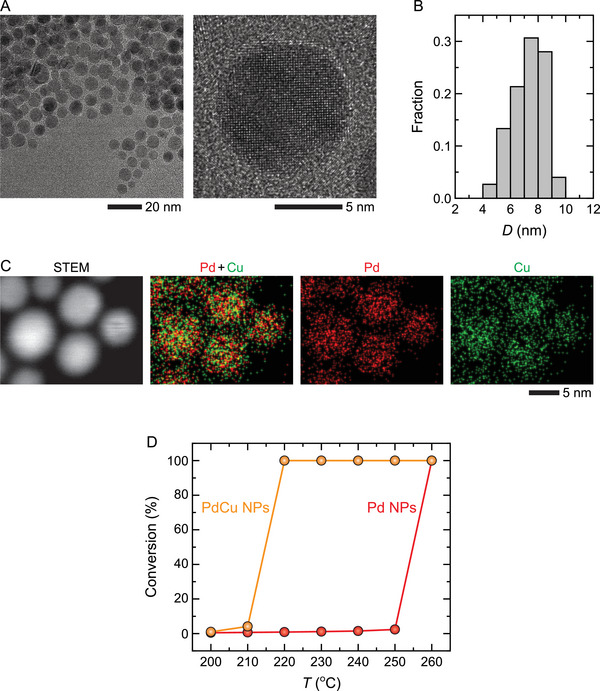
PdCu alloy NPs. A) TEM images and B) size distribution of the as‐synthesized PdCu NPs. The average diameter of the NPs is *D* = 7 ± 1 nm. C) STEM and corresponding energy‐dispersive X‐ray spectroscopy (EDX) images of the NPs. D) Temperature‐dependent catalytic performance of the PdCu and Pd NPs during CO oxidation reaction in an atmosphere of $p_{\mathrm{CO}}/p_{O_{2}}\approx 0.5$ (760 Torr of 9% CO, 18% O_2_, and 73% N_2_) at 200–260 °C. Both of the catalyst samples were calcined in pure N_2_ (760 Torr of N_2_) at 200 °C for 1 h before the CO oxidation reaction.

The as‐synthesized alloy PdCu NPs are spherical single‐crystalline NPs with an average diameter of 7 nm (**Figure** **1**A,B). As shown in Figure 1C, Pd (50% (at.)) and Cu (50% (at.)) are distributed uniformly within the NPs. To evaluate the catalytic performance of the PdCu NPs, we assessed the CO‐to‐CO_2_ conversion during the CO oxidation reaction in a conventional flow reactor and compared the conversion rate to that of pure Pd NPs (Figure 1D). PdCu NPs reach a 100% CO conversion rate at a lower temperature than Pd NPs (220 °C for PdCu vs 260 °C for Pd), indicating that the presence of Cu atoms in the alloy NPs improves their catalytic activity significantly.

**Figure 2:**
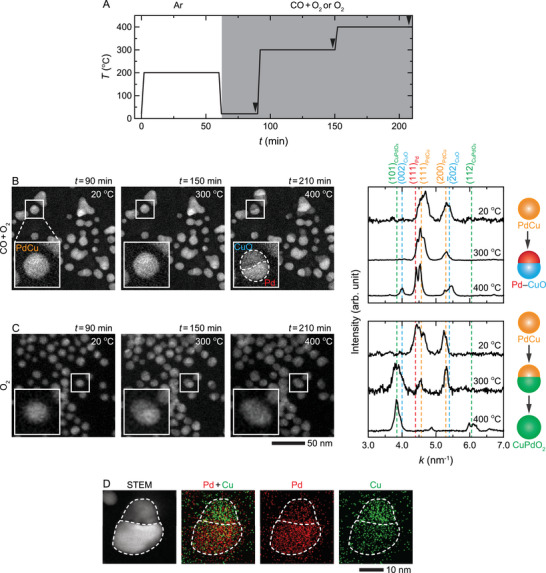
Oxidation of the PdCu alloy NPs. A) Heating profile for the in situ studies shown in (B,C). First, the NPs were annealed in pure Ar atmosphere (760 Torr of Ar) at 200 °C for 60 min to remove the residual surfactants. After cooling the NPs back to room temperature (20 °C), and as shown in (B,C), either O_2_ (20% O_2_ and 80% He) or a mixture of CO and O_2_ ($p_{\mathrm{CO}}/p_{O_{2}}\approx 0.5$, i.e., 9% CO, 18% O_2_, and 73% He) were introduced at atmospheric pressure and the reaction was allowed to proceed first at 300 °C and then at 400 °C for 60 min each. The black arrows correspond to the time points of the STEM image series and electron diffraction profiles shown in (B,C). STEM images and electron diffraction profiles of the NPs during B) CO oxidation reaction at $p_{\mathrm{CO}}/p_{O_{2}}\approx 0.5$ and C) oxidation in an O_2_ environment. The dashed vertical lines in the diffraction profiles correspond to the diffraction peaks of PdCu (orange), CuO (blue), Pd (red), and CuPdO_2_ (green) (Section S1, Supporting Information). Figure S10 (Supporting Information) shows the original electron diffraction images used to generate the diffraction profiles in (B,C). D) STEM and corresponding EDX images showing a PdCu alloy NP after its phase segregation during the CO oxidation reaction.

To identify the structural origin for the enhancement in the activity of PdCu NPs, we tracked the evolution of the NPs during a CO oxidation reaction with operando TEM. First, we annealed the NPs in pure Ar (760 Torr of Ar) in a gas cell to remove the residual surfactants (as we did in the case of flow reactor studies shown in Figure 1D), and then we introduced the O_2_‐rich reactive gas with $p_{\mathrm{CO}}/p_{O_{2}}\approx 0.5$ (760 Torr of 9% CO, 18% O_2_, and 73% He) (**Figure** **2**A). Scanning TEM (STEM) images show that the NPs remained unchanged at 300 °C, but at 400 °C, the STEM contrast across the NPs changed, showing that one side of the NPs was brighter than the other (Figure 2B). This change in the contrast is due to the phase separation of Pd and Cu in the NPs (Figure 2D). Here, the most plausible explanation is that under the O_2_‐rich atmosphere, the alloy NPs segregated into Pd–CuO NPs via selective oxidation of Cu. The presence of CuO and Pd is confirmed by the appearance of the respective diffraction peaks at 400 °C (Figure 2B).

To establish that the PdCu NPs transformed into Pd–CuO during the CO oxidation reaction because of O_2_, we tested the NPs in a pure O_2_ environment (760 Torr of 20% O_2_ and 80% He). The STEM contrast of the individual NPs is again split into brighter and slightly darker regions after heating the NPs at 300 °C for 60 min in the pure O_2_ environment (Figure 2C: *t* = 150 min). While the STEM contrast of these NPs went back to being uniform at 400 °C, there is a notable increase in their sizes in comparison to their original shapes (Figure 2C: *t* = 210 min vs *t* = 90 min). The observed contrast change and enlargement of the PdCu NPs at 300–400 °C are consistent with their transformation into CuPdO_2_ NPs, as confirmed by their diffraction profiles (Figure 2C) and high‐resolution TEM images (Section S2, Supporting Information).

Observed differences in the oxidation of the PdCu NPs under two different atmospheres hint at two different oxidation states of Pd atoms (i.e., Pd^0^ in CO and O_2_ vs Pd^2+^ in O_2_). In fact, density functional theory (DFT) calculations show that bulk PdO is readily reduced to metallic Pd by CO adsorption, indicating that PdO is preferred to metallic Pd only under a pure O_2_ environment.^[^ ^29^ ^]^ In contrast to Pd, Cu is oxidized both during the CO oxidation reaction^[^ ^30^ ^]^ and under a pure O_2_ atmosphere.^[^ ^31^ ^]^ Moreover, the extent of the observed transformation of PdCu NPs into Pd–CuO NPs during the CO oxidation reaction under relevant atmospheric pressure, as shown in Figure 2B, differs drastically from earlier studies conducted at much lower reaction pressures (<1 Torr).^[^ ^1^, ^13^ ^]^ Because of the reactions being conducted under low pressures, the earlier studies found the NP to have subnanometer surface oxides. Hence, to establish relevant structures of nanocatalysts, it is important to explore them under real reactive conditions.

**Figure 3:**
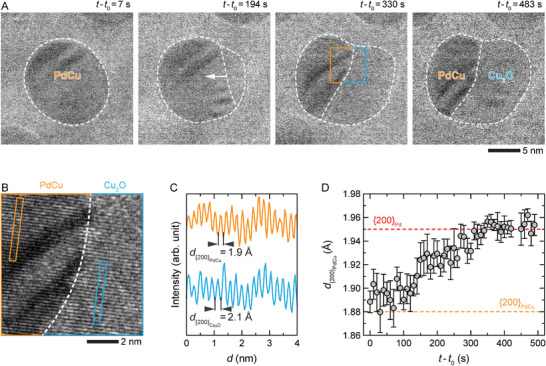
Selective oxidation of Cu in a PdCu NP during CO oxidation reaction. A) In situ TEM image series showing the oxidation of the NP in an atmosphere of $p_{\mathrm{CO}}/p_{O_{2}}\approx 0.5$ (760 Torr of 9% CO, 18% O_2_, and 73% He) at 400 °C (Video S1, Supporting Information). The white arrow indicates the direction of the PdCu–Cu_2_O interface movement, and *t*_0_ is the time point at which the NP was heated to 400 °C. B) Magnified view of the PdCu–Cu_2_O interface (dashed white curve) taken from the orange–blue box region in (A). C) Line profiles obtained from orange and blue boxes in (B) show that the respective lattice planes correspond to {200}_PdCu_ (orange) and ${\left\{200\right\}}_{{\mathrm{Cu}}_{2}O}$ (blue). D) Plot of $d_{{\left\{200\right\}}_{\mathrm{PdCu}}}$ during the CO oxidation reaction as NP underwent segregation (see Section S3, Supporting Information for the details of the analysis).

To resolve nanoscale details through which Cu and Pd in the PdCu NPs separate during the CO oxidation reaction, we tracked the NP evolution at 400 °C with TEM (**Figure** **3**). The Cu_2_O formed at the right side of the single‐crystalline NP and grew into the alloy region until half of the NP was oxidized (Figure 3A–C). As Cu oxidized, the fraction of Pd in the alloy region of the NP increased, as seen by the increase in lattice spacing (Figure 3D), which is consistent with Pd enrichment. Furthermore, segregated Cu_2_O gradually transformed into CuO over time, as shown in Figure 2B, indicating that Cu_2_O is an intermediate oxide that forms during the reaction. Moreover, the reason for the apparent segregation of the Cu_2_O region from the rest of the NP, as seen in Figure 3A, is due to the preferential adsorption or diffusion of O_2_ toward the existing oxide regions rather than nucleating new oxide sites.^[^ ^32^, ^33^, ^34^ ^]^

**Figure 4:**
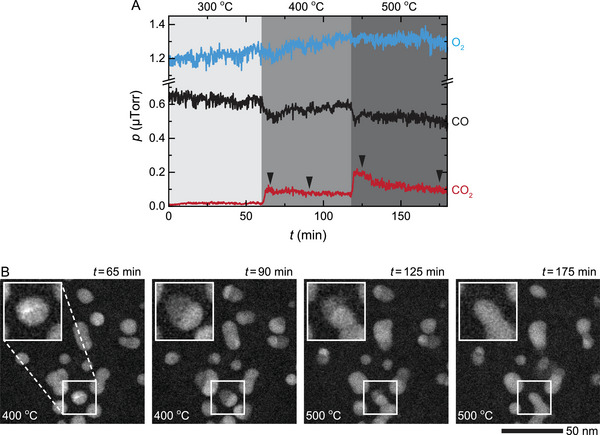
Oxidation and reduction of the PdCu NPs during CO oxidation reaction. A) Changes in gas compositions during the CO oxidation reaction in an atmosphere of $p_{\mathrm{CO}}/p_{O_{2}}\approx 0.5$ (760 Torr of 9% CO, 18% O_2_, and 73% He) at 300–500 °C. The arrows correspond to the time points of the image series shown in (B). B) In situ STEM image series show that the PdCu NPs oxidized into Pd−CuO NPs at 400 °C (*t* = 65–90 min), and then they reduced back into PdCu NPs at 500 °C (*t* = 125–175 min).

**Figure 5:**
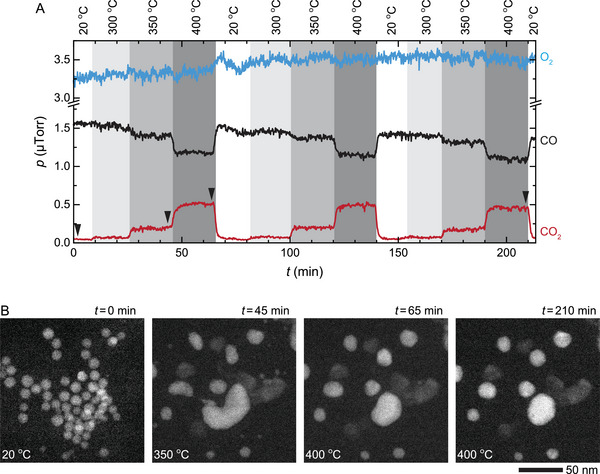
Three cycles of CO oxidation reaction over PdCu NPs. A) Change in gas compositions during the CO oxidation reaction in an atmosphere of $p_{\mathrm{CO}}/p_{O_{2}}\approx 0.5$ (760 Torr of 9% CO, 18% O_2_, and 73% He) at 20–400 °C. The reaction was held at each temperature (i.e., 300, 350, and 400 °C) for ≈20 min. The black arrows correspond to the time points of the image series shown in (B). B) In situ STEM image series of the NPs during the reaction described in (A).

It is natural to expect that the structural changes in the NPs taking place during the reaction should impact their catalytic performance. To test whether this is the case or not, we tracked the structure of individual PdCu NPs along with their activity during the CO oxidation reaction in operando (**Figure** **4**). At a temperature of 400 °C and an atmosphere of $p_{\mathrm{CO}}/p_{O_{2}}\approx 0.5$, the NPs transformed into active Pd−CuO NPs and produced CO_2_ (Figure 4A,B: *t* = 65–90 min). However, once the temperature was increased to 500 °C, the production of CO_2_ declined gradually (Figure 4A: *t* = 120–180 min), which coincided with the transformation of Pd−CuO NPs back into their metallic PdCu form as seen from STEM images (i.e., segregated contrast at *t* = 125 min changed back to uniform contrast at *t* = 175 min as shown in Figure 4B). The fact that the presence of CuO region promotes the reaction is in line with Pd–CuO interface being less susceptible to CO poisoning than pure Pd surface,^[^ ^35^ ^]^ because O_2_ adsorbs stronger to Pd atoms at the interface perimeter. This selective O_2_ adsorption onto the Pd atoms is due to the increase in the adsorption energy caused by the electron transfer from the oxide to interfacial Pd atoms.^[^ ^35^, ^36^ ^]^ More notably, our in operando test of three consecutive reaction cycles at 20–400 °C confirms that both the activity and morphology of Pd−CuO NPs remain stable (**Figure** **5**A,B: *t* = 65 min vs *t* = 210 min). Note that while the temperature needed to initiate the catalytic reaction during operando TEM studies is higher (e.g., absence of CO conversion at 300 °C in Figures 4 and 5) compared to the reaction in the conventional flow reactor (Figure 1D), it can be easily explained by the short residence time of the gas molecules flowing over the NPs in the gas cell during TEM imaging.^[^ ^25^ ^]^

To establish that our findings are not specific only to PdCu NPs but also applicable to other Cu‐based bimetallic NPs, we extended our study to RhCu NPs (Section S5, Supporting Information). Similar to the PdCu NPs (Figures 2, 5), in the reactive gas environment of $p_{\mathrm{CO}}/p_{O_{2}}\approx 0.5$ and at a temperature range of 300–400 °C, Cu oxidized into CuO, and the NPs transformed into very active and stable Rh–CuO NPs (Figures S7B,D and S8, Supporting Information). These results not only confirm that the RhCu NPs behave similarly to the PdCu NPs but also suggest that the oxidation of the non‐noble component (in this case, the formation of CuO regions) improves the catalytic activity in general.

## 3 Conclusion

Our in situ observations suggest that the selective oxidation of a non‐noble metallic component in bimetallic alloy nanocatalysts such as PdCu and RhCu alloy NPs can significantly improve their catalytic activity in contrast to the monometallic noble metal NPs (i.e., Pd or Rh). Furthermore, the structure and reactivity of the transformed NPs, which arises from the segregation of the alloy into non‐noble metal oxide and noble metal components during the reaction, remain stable during multiple cycles under normal reaction conditions. More broadly, taking a direct approach to observing the reactions and identifying active catalytic structures, as described here, can play an important role in the design and improvement of future nanocatalysts.

## 4 Experimental Section

### Materials

The following reagents from Sigma–Aldrich Co. (St Louis, MO, USA) were used to synthesize the NPs: sodium tetrachloropalladate(II) (Na_2_PdCl_4_, Cat. No. 379808), copper(II) acetylacetonate (Cu(acac)_2_, Cat. No. 514365), rhodium(III) acetylacetonate (Rh(acac)_3_, Cat. No. 282774), rhodium(III) chloride hydrate (RhCl_3_, Cat. No. 206261), d‐(−)‐ribose (C_5_H_10_O_5_, Cat. No. R7500), silica (SiO_2_, Cat. No. S5505), sodium borohydride (NaBH_4_, Cat. No. 452882), acetone (C_3_H_6_O, Cat. No. 650101), 1‐octadecene (ODE, C_18_H_36_, Cat. No. O806), and oleylamine (OAm, C_18_H_37_N, Cat. No. O7805). The following solvents were used to wash the NPs: hexane (C_6_H_14_, Cat. No. 32293, Sigma–Aldrich Co., St Louis, MO, USA) and ethanol (C_2_H_6_O, Cat. No. 10437341, Fisher Scientific U.K. Ltd, Loughborough, UK). Deionized (DI) water with a resistivity of 18.2 MΩ cm was used to prepare all aqueous solutions used in this study.

### Synthesis of PdCu and Pd NPs

The PdCu alloy NPs were synthesized based on a modified procedure of Tong et al.^[^ ^37^ ^]^ Specifically, 5 mg of Na_2_PdCl_4_, 7 mg of Cu(acac)_2_, 8 mg of ribose, 1.3 mL of ODE, and 1.3 mL of OAm were mixed in a 20 mL capped glass vial and ultrasonicated for 30 min to dissolve the solid chemicals (Na_2_PdCl_4_, Cu(acac)_2_, and ribose) in the mixed solvent (ODE and OAm). Next, the vial containing this homogeneous solution was heated from room temperature to 180 °C in an oil bath and kept at this temperature for 180 min, during which the color of the final solution turned black. The synthesized NPs were washed five times in a hexane/ethanol mixture (prepared at a 1:1 volume ratio) by centrifugation and then dispersed in hexane before use. The Pd NPs were synthesized following the same procedures as the PdCu NPs except, in this case, the precursor solution contained 8 mg of Na_2_PdCl_4_, 8 mg of ribose, 1.3 mL of ODE, and 1.3 mL of OAm.

### Synthesis of RhCu NPs

10 mg of Rh(acac)_3_, 7 mg of Cu(acac)_2_, 18 mg of ribose, 1.3 mL of ODE, and 1.3 mL of OAm were mixed in a 20 mL capped glass vial and ultrasonicated for 30 min to dissolve the solid chemicals (Rh(acac)_3_, Cu(acac)_2_, and ribose) in the mixed solvent (ODE and OAm). Then the solution was heated from room temperature to 180 °C in an oil bath and kept at this temperature for 120 min, during which the color of the final solution turned black. The synthesized NPs were washed three times in the hexane/ethanol mixture (prepared at a 1:1 volume ratio) by centrifugation and then dispersed in hexane before use.

### Synthesis of Rh/SiO_2_ Catalysts

The Rh/SiO_2_ catalysts shown in FigureS9 (Supporting Information) were synthesized based on the modified procedures of Akbayrak et al.^[^ ^38^ ^]^ Specifically, 8 mg of RhCl_3_, 100 mg of SiO_2_, and 90 mL of DI water were mixed in a flask. The mixed solution was stirred at room temperature for 30 min to disperse the SiO_2_ powder uniformly in the solution. Then 10 mL of 10 mm NaBH_4_ aqueous solution was added into the mixed solution dropwise, and the solution was stirred for another 30 min. The catalysts were washed three times in DI water by centrifugation and dried in a vacuum chamber overnight. The final metal loading of the Rh/SiO_2_ catalyst was 3% (wt.) (measured with inductively coupled plasma‐optical emission spectrometry (ICP‐OES)).

### Measurements of Catalytic Activity

The as‐synthesized NPs (PdCu, Pd, and RhCu NPs) and SiO_2_ powder were dispersed in 15 mL of hexane, respectively, and stirred for 2 h to facilitate the adsorption of NPs onto SiO_2_. The supported catalysts were centrifuged and washed twice in acetone and further dried in a vacuum chamber overnight. The final metal loadings were maintained at 3% (wt.) for PdCu/SiO_2_, Pd/SiO_2_, and RhCu/SiO_2_ catalysts (measured with ICP‐OES).

20 mg of each as‐prepared catalyst samples (i.e., PdCu/SiO_2_, Pd/SiO_2_, RhCu/SiO_2_, or Rh/SiO_2_) were immobilized by the quartz wool and loaded into a quartz tube. Then the quartz tube was inserted into a Carbolite MTF 9/15/130 tube furnace (Carbolite Gero Ltd., Sheffield, UK) and connected to the stainless steel pipes at both ends. The inlet pipe was connected to the Brooks 5850E mass flow controllers (Brooks Instrument, Hatfield, PA, USA). The outlet pipe was connected to the Agilent 7890A gas chromatograph (Agilent Technologies Inc., Santa Clara, CA, USA). The catalyst sample was first calcined in pure N_2_ at 200 °C for 1 h; then, the reaction gas (760 Torr of 9% CO, 18% O_2_, and 73% N_2_) was introduced into the quartz tube to measure the catalytic activity. The gas flow rate was kept at 40 mL min^−1^. The conversion rate at each temperature was obtained by averaging three steady readings from the gas chromatograph, and the temperature at which CO conversion reaches 100% was verified twice for all the tested catalyst samples.

### In Situ TEM Experiments

A Thermofisher Titan (S)TEM (Thermo Fisher Scientific Ltd., Hillsboro, OR, USA) equipped with a Bruker Xflash 6T|30 EDX spectrometer (Bruker, Billerica, MA, USA) was used for in situ TEM studies. The (S)TEM was operated with an accelerating voltage of 300 kV. During the in situ STEM experiments shown in Figures 2, 4, and 5, and Figures S5, S7, and S8 (Supporting Information), the NPs were only exposed to the electron beam (≈60 e^−^ Å^−2^ s^−1^) at the indicated time points to acquire the displayed image frames. In situ TEM image series shown in Figure 3, Figures S2 and S6 (Supporting Information) were acquired with a Gatan K2 IS camera (Gatan Inc., Pleasanton, CA, USA) with an electron flux of 100−200 e^−^ Å^−2^ s^−1^.

A DENSsolutions Climate TEM holder (DENSsolutions, Delft, Netherlands) was used for all in situ TEM studies of the NPs in the gaseous environments while heating. Before the in situ TEM studies, 5 µL of the PdCu or RhCu NP solution was drop‐casted onto the bottom chip of a gas cell (DENSsolutions, Delft, Netherlands) and allowed to dry. Then, the gas cell was assembled inside the holder (with connected inlet and outlet tubings) and tested for any potential leaks before inserting the holder into the TEM. For all in situ TEM studies, gases were introduced into the gas cell at a flow rate of 40−50 µL min^−1^ and at a pressure of 760 Torr using the DENSsolutions gas delivery system. Simultaneously, the outlet gas from the holder was analyzed with a quadrupole mass spectrometer (Stanford Research Systems, Sunnyvale, CA, USA), and the pressure of the gas going through the analyzer chamber was about 10^−5^ Torr.

### Extraction of the Electron Diffraction Profiles from TEM Diffraction Images

The radial diffraction profiles shown in Figure 2B,C and Figures S4B, S5B, and S7B,C (Supporting Information) are the plots of radial variation coefficient $\tilde{\sigma }\left(k\right)$ obtained from electron diffraction images:^[^ ^22^, ^39^ ^]^

(1) $$\tilde{\sigma }\left(k\right)=\frac{\sqrt{\bar{I{\left(k\right)}^{2}}-{\bar{I\left(k\right)}}^{2}}}{\bar{I\left(k\right)}}$$

Here, *k* is the outward distance measured from the center of the diffraction pattern (i.e., radius), and *I*(*k*) is the intensity of the diffraction pattern at the distance *k*. First, the central position of the diffraction pattern in the image was identified manually. Then the pixel intensities of the image were collected, and the variation coefficient $\tilde{\sigma }\left(k\right)$ was computed as a function of *k*. These image processing codes were written in Java.^[^ ^40^ ^]^

## Conflict of Interest

The authors declare no conflict of interest.

## Supporting information

Supporting Information

Supplemental Video 1

Supplemental Video 2

Supplemental Video 3

## Data Availability

The data that support the findings of this study are available from the corresponding author upon reasonable request.
