# Supplementary material for: Phase Segregation in PdCu Alloy Nanoparticles During CO Oxidation Reaction at Atmospheric Pressure
Source: Adv Sci (Weinh). 2023 Jun 28;10(25):2302663. doi: 10.1002/advs.202302663 (PMC10477843; doi:10.1002/advs.202302663)
Supplement: Supplementary file 1 — Supporting Information [file ADVS-10-2302663-s002.pdf]

ADVANCED SCIENCE
Open Access

Supporting Information

for *Adv. Sci.*, DOI 10.1002/advs.202302663

Phase Segregation in PdCu Alloy Nanoparticles During CO Oxidation Reaction at Atmospheric Pressure

*Yingying Jiang, Alvin M. H. Lim, Hongwei Yan, Hua Chun Zeng and Utkur Mirsaidov**

ADVANCED SCIENCE
Open Access

Supporting Information

for *Adv. Sci.*, DOI 10.1002/advs.202302663

Phase Segregation in PdCu Alloy Nanoparticles During CO Oxidation Reaction at Atmospheric Pressure

*Yingying Jiang, Alvin M. H. Lim, Hongwei Yan, Hua Chun Zeng and Utkur Mirsaidov**

Supporting Information

Phase Segregation in PdCu Alloy Nanoparticles during CO Oxidation Reaction at Atmospheric Pressure

*Yingying Jiang[1, 2], Alvin M. H. Lim[3], Hongwei Yan[1, 2], Hua Chun Zeng[3], and Utkur Mirsaidov[1, 2, 4, 5]**

1. Department of Physics, National University of Singapore, Singapore, 117551, Singapore
2. Centre for BioImaging Sciences, Department of Biological Sciences, National University of Singapore, Singapore, 117557, Singapore
3. Department of Chemical and Biomolecular Engineering, National University of Singapore, Singapore, 119260, Singapore
4. Centre for Advanced 2D Materials and Graphene Research Centre, National University of Singapore, Singapore, 117546, Singapore
5. Department of Materials Science and Engineering, National University of Singapore, Singapore, 117575, Singapore

*Correspondence: mirsaidov@nus.edu.sg

Table of Contents

1. Structures of metals and oxides 1
2. PdCu NPs in the O_2 gas environment 2
3. Estimating the lattice plane spacings of PdCu NPs 3
4. CO oxidation reaction over PdCu NPs in an atmosphere of $p_{CO}/p_{O_2} \approx 1$ 4
5. RhCu NPs 5
6. Electron diffraction images of PdCu and RhCu NPs during reactions 8
7. Supporting video captions 9
8. Supporting references 9

1. Structures of metals and oxides

The standard lattice plane spacings of Pd (JCPDS no. 65-2867), CuO (JCPDS no. 45-0937), and $CuPdO_2$ (JCPDS no. 44-0185) are listed in Table S1. $CuPdO_2$ has a tetragonal crystal structure (Figure S1A), and CuO has a monoclinic crystal structure (Figure S1B). The lattice plane spacings of PdCu alloy shown in Table S1 were estimated by summing the corresponding Pd and Cu (JCPDS no. 65-9026) lattice spacings proportionally (*i.e.*, 50% (at.) of Pd and 50% (at.) of Cu). The reason for this is that according to Vegard's law, the unit cell parameters vary linearly with the composition of a solid solution.[1]

Table S1. Lattice plane spacings of Pd, PdCu, CuO, and $CuPdO_2$.

Pd		PdCu		CuO		$CuPdO_2$	
hkl	d_{hkl} (Å)	*hkl*	d_{hkl} (Å)	*hkl*	d_{hkl} (Å)	*hkl*	d_{hkl} (Å)
111	2.25	111	2.17	$\bar{1}10$	2.75	002	2.63
200	1.95	200	1.88	002	2.53	101	2.59
220	1.38	220	1.33	111	2.32	110	2.10
311	1.17	311	1.13	200	2.31	112	1.64
222	1.12	222	1.08	$\bar{2}02$	1.87	103	1.51

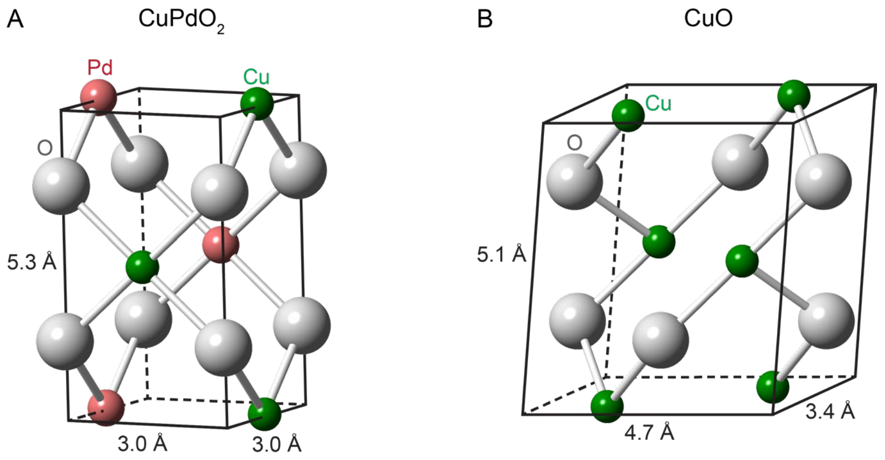

Figure S1. Crystal structures of $CuPdO_2$ and CuO. The unit cells of (**A**) $CuPdO_2$, and (**B**) CuO. These atomic models were drawn using CrystalMaker® 9.0.3 package.[2]

2. PdCu NPs in the O_2 gas environment

Our *in situ* TEM observations of PdCu alloy NPs in an O_2 environment (760 Torr of 20% O_2 and 80% He) at 300 °C show that $CuPdO_2$ grows on the NP by simultaneous oxidation of Pd and Cu (Figure S2A−B). The growth of $CuPdO_2$ proceeds via the movement of a PdCu–$CuPdO_2$ interface (Figure S2C). At 400 °C, these NPs were fully oxidized and transformed into single-crystalline or polycrystalline $CuPdO_2$ NPs (Figure S3).

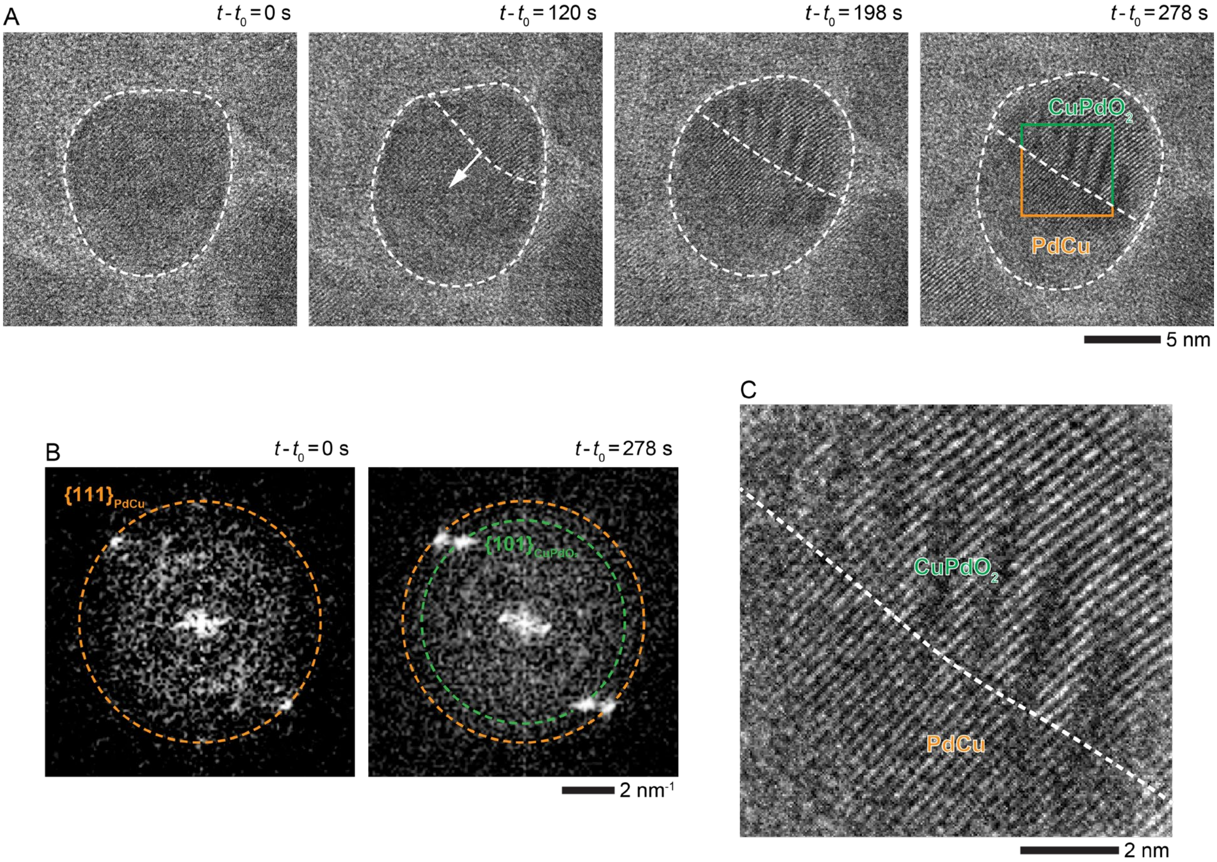

Figure S2. Oxidation of a PdCu NP in an O_2 environment. (**A**) Oxidation of the NP in an O_2 environment (760 Torr of 20% O_2 and 80% He) at 300 °C (Supporting Video 2). The white arrow indicates the direction of the PdCu–$CuPdO_2$ interface movement. Here, t_0 is the time point at which we started recording the process. (**B**) FFTs of two image frames displayed in (A). The dashed orange and green circles correspond to the reciprocals of PdCu and $CuPdO_2$ lattice plane spacings, respectively (*i.e.*, $k_{\{111\}_{PdCu}}$ = 4.6 nm^{-1}, and $k_{\{101\}_{CuPdO_2}}$ = 3.9 nm^{-1}). (**C**) Magnified view of the PdCu–$CuPdO_2$ interface (dashed white curve) from the orange–green box region in (A).

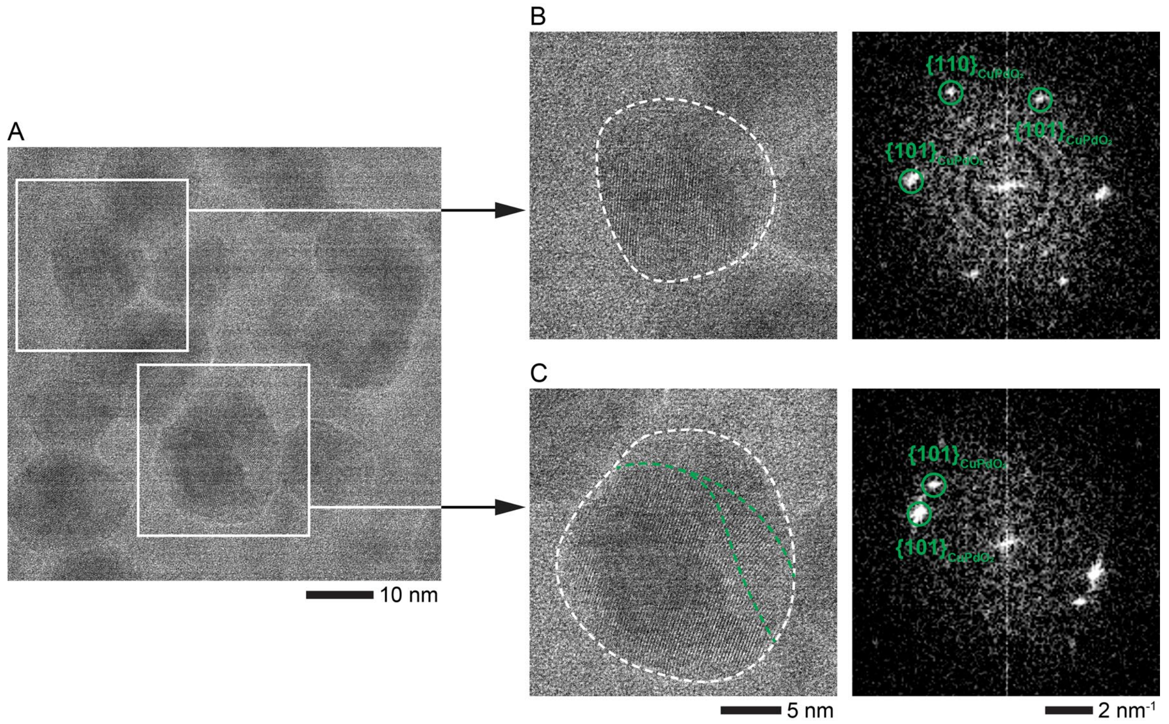

Figure S3. Structures of the $CuPdO_2$ NPs. (**A**) TEM image of PdCu NPs after their oxidation in an O_2 environment (760 Torr of 20% O_2 and 80% He) at 400 °C for 60 min. (**B**–**C**) Magnified views and corresponding FFTs of two NPs shown in (A). The dashed green curves in (C) highlight the grain boundaries in the polycrystalline $CuPdO_2$ NP.

3. Estimating the lattice plane spacings of PdCu NPs

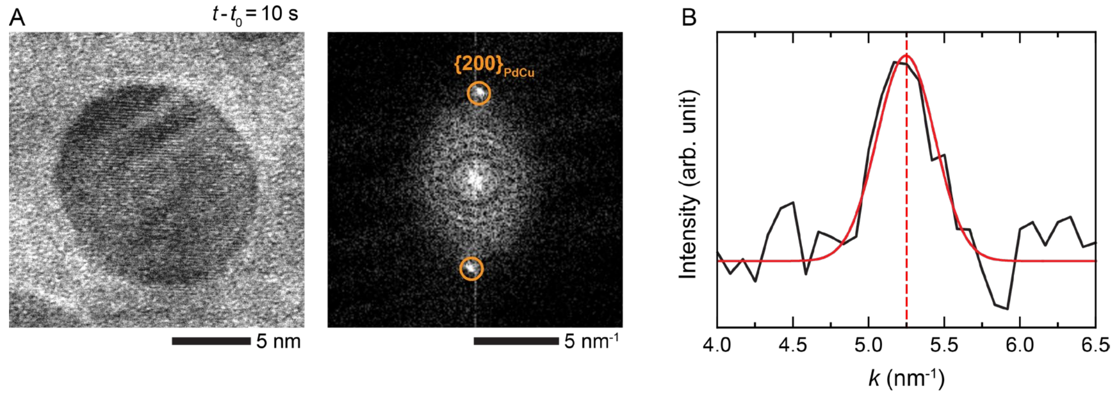

Figure S4. Measurement of the lattice plane spacing in a PdCu NP from a TEM image. (**A**) TEM image and its FFT of the PdCu NP displaying $\{200\}_{\mathrm{PdCu}}$ lattice spacings. (**B**) Radial profile (black curve) extracted from the FFT in (A) and the corresponding Gaussian fit (red curve). The dashed red line is the center position of the Gaussian peak.

To track the subtle changes in $d_{\{200\}_{\mathrm{PdCu}}}$ during the segregation of Cu from the alloy NP (Figure 3A), we first drift-corrected *in situ* TEM image series, which were recorded at a rate of 5 frames per second. We summed every consecutive 50 frames to enhance the image contrast of the NP and then obtained FFTs from these summed images (Figure S4A). Next, we extracted

the radial profiles of the FFTs and fitted them with the Gaussian function (Figure S4B). The center position of the Gaussian peaks represent $d_{\{200\}_{PdCu}}$ in the reciprocal space at a given time point (Figure 3D).

4. CO oxidation reaction over PdCu NPs in an atmosphere of $p_{CO}/p_{O_2} \approx 1$

To see how PdCu NPs change under different p_{CO}/p_{O_2} conditions, we also imaged the NPs at $p_{CO}/p_{O_2} \approx 1$ (760 Torr of 17% CO, 17% O_2, and 66% He) (Figure S5). As seen from Figure S5A–B, Cu in the PdCu NPs segregated by forming CuO at 300–400 °C. Here, while the amount of CuO was significantly less than under the O_2-rich case ($p_{CO}/p_{O_2} \approx 0.5$) shown in Figure 2B, the way the segregation proceeded was similar to that of under the O_2-rich environment (Figure S6 *vs.* Figure 3). The NPs had stable activity during the CO oxidation reaction at 400–500 °C (Figure S5C: t = 160–270 min).

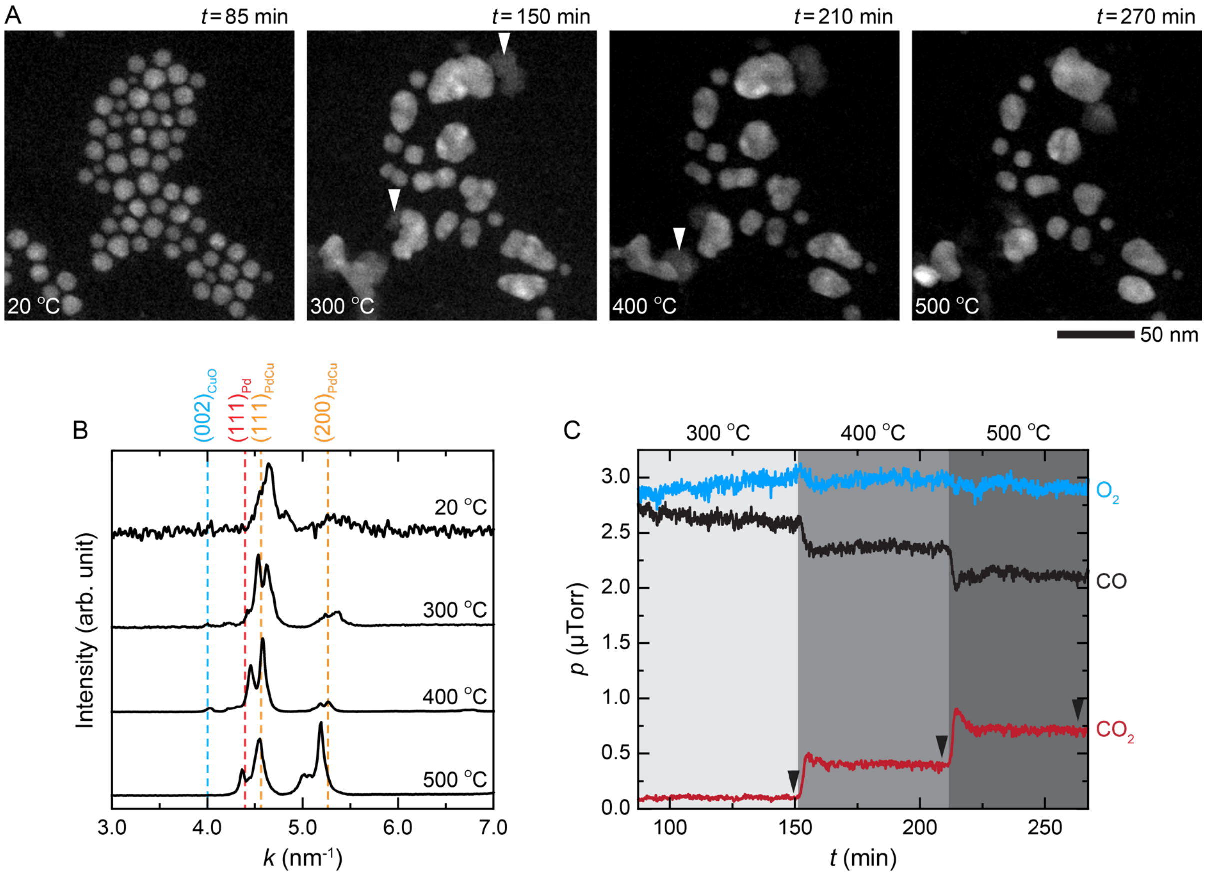

Figure S5. CO oxidation reaction over PdCu NPs in an atmosphere of $p_{CO}/p_{O_2} \approx 1$. (**A**) STEM images and (**B**) electron diffraction profiles of the NPs during the CO oxidation reaction in an atmosphere of $p_{CO}/p_{O_2} \approx 1$ (760 Torr of 17% CO, 17% O_2, and 66% He) at 20–500 °C. The reaction was held at each temperature (*i.e.*, 300, 400, and 500 °C) for 60 min. The white arrows in (A) point to the CuO regions of the NPs that formed during the CO oxidation reaction. The vertical dashed lines in (B) correspond to the diffraction peaks of PdCu (orange), CuO (blue), and Pd (red). (**C**) Changes in gas composition during the CO oxidation reaction at 300–500 °C. The black arrows correspond to the time points of the image series shown in (A).

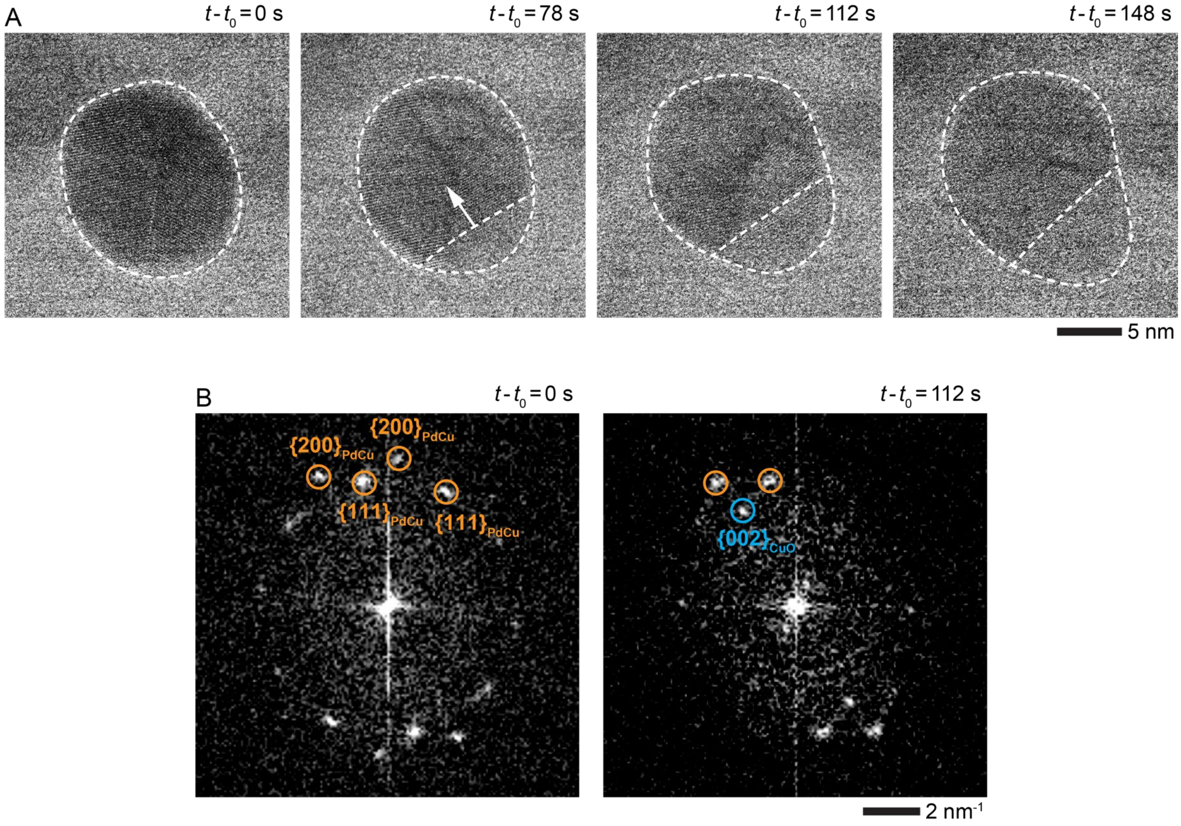

Figure S6. Selective oxidation of Cu in a PdCu NP during CO oxidation reaction at $p_{CO}/p_{O_2} \approx 1$. (**A**) Oxidation of the NP in an atmosphere of $p_{CO}/p_{O_2} \approx 1$ (760 Torr of 17% CO, 17% O_2, and 66% He) at 400 °C (Supporting Video 3). The white arrow indicates the direction of the PdCu–CuO interface movement. Here, t_0 is the time point at which we started recording the process. (**B**) The FFTs of two image frames displayed in (A).

5. RhCu NPs

To confirm that our results for PdCu NPs extend to other Cu-based alloy nanocatalysts, we performed similar studies using RhCu NPs. Figure S7A shows the STEM and EDX images of the as-synthesized RhCu NPs with the corresponding elemental compositions of 70 and 30% (at.) for the Cu core and Rh shell, respectively. In the reactive gas environment of $p_{CO}/p_{O_2} \approx 0.5$ (760 Torr of 9% CO, 18% O_2, and 73% He) and at a temperature range of 300–400 °C, only Cu was oxidized while Rh remained metallic, and the NPs transformed into active Rh–CuO NPs (Figures S7B, D). However, in a pure O_2 environment (760 Torr of 20% O_2 and 80% He), both Cu and Rh oxidize, and the NPs transformed into Rh_2O_3–CuO (Figure S7C).

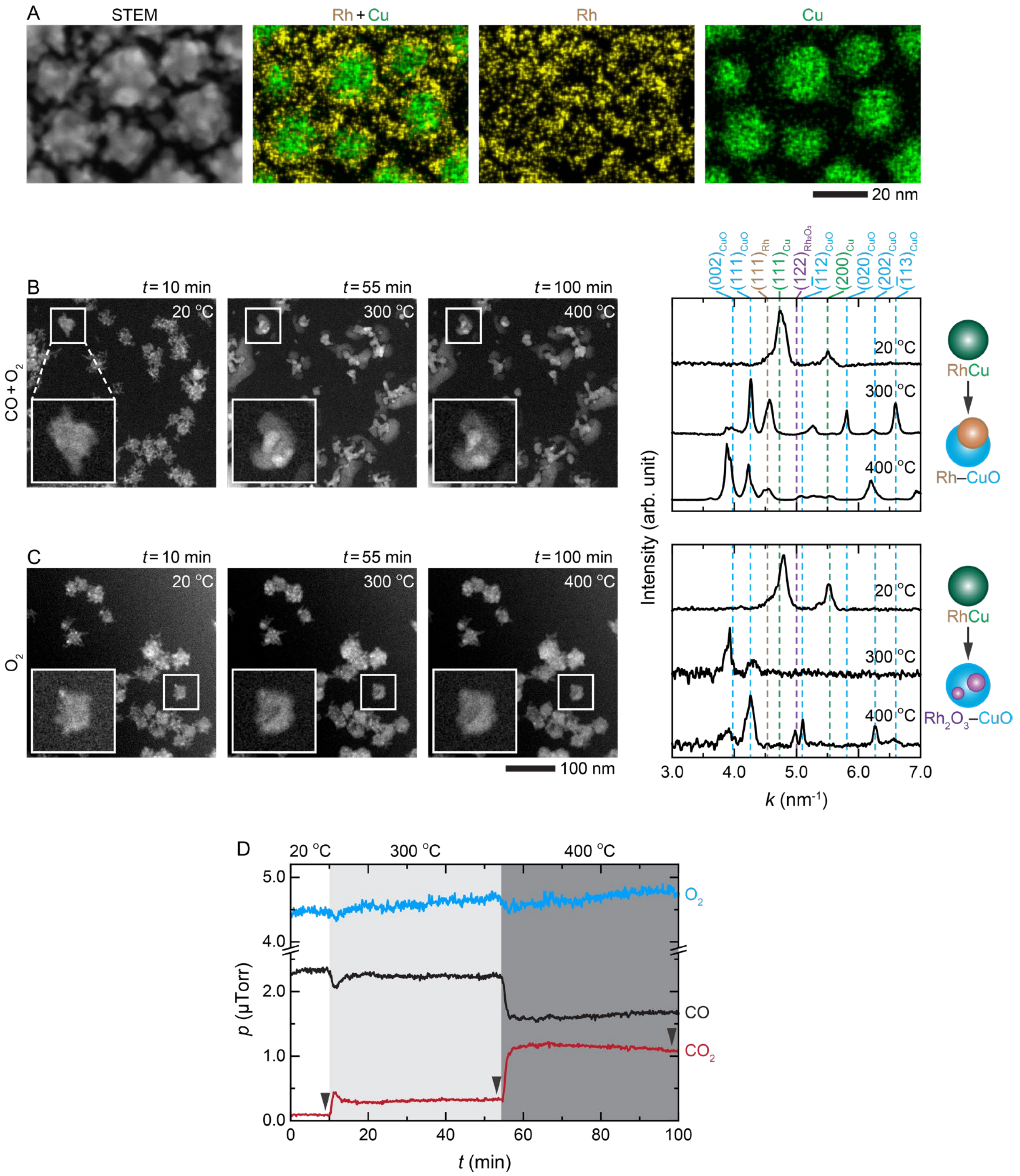

Figure S7. Oxidation of the RhCu NPs. (**A**) STEM and corresponding EDX images of the as-synthesized RhCu NPs. *In situ* STEM image series and corresponding electron diffraction profiles of the NPs during (**B**) CO oxidation reaction in an atmosphere of $^{p_{CO}}/_{p_{O_2}} \approx 0.5$ (760 Torr of 9% CO, 18% O_2, and 73% He) and (**C**) oxidation in an O_2 environment (760 Torr of 20% O_2 and 80% He). The reaction was held at each temperature (*i.e.*, 300 and 400 °C) for 45 min. The dashed lines in the plots of diffraction profiles shown in (B–C) correspond to the diffraction peaks of Rh (brown), Cu (green), CuO (blue), and Rh_2O_3 (purple). (Figure S11 displays the original electron diffraction images used to generate these profiles). (**D**) Change in gas compositions during the CO oxidation reaction at 20–400 °C and $^{p_{CO}}/_{p_{O_2}} \approx 0.5$. The arrows in the plot correspond to the time points of the image series and electron diffraction profiles shown in (B).

To study the stability of the RhCu alloy NPs during the reaction, we monitored the NPs during three reaction cycles at 20–400 °C (Figure S8). The NPs started exhibiting catalytic activity at 300 °C (Figure S8A: *t* = 10–30 min), with the Cu segregation occurring simultaneously during the first cycle (Figure S8B: *t* = 25 min). In all three cycles, the production of CO_2, as well as the structure of the segregated NPs, remained stable (Figure S8A–B: *t* = 25–200 min).

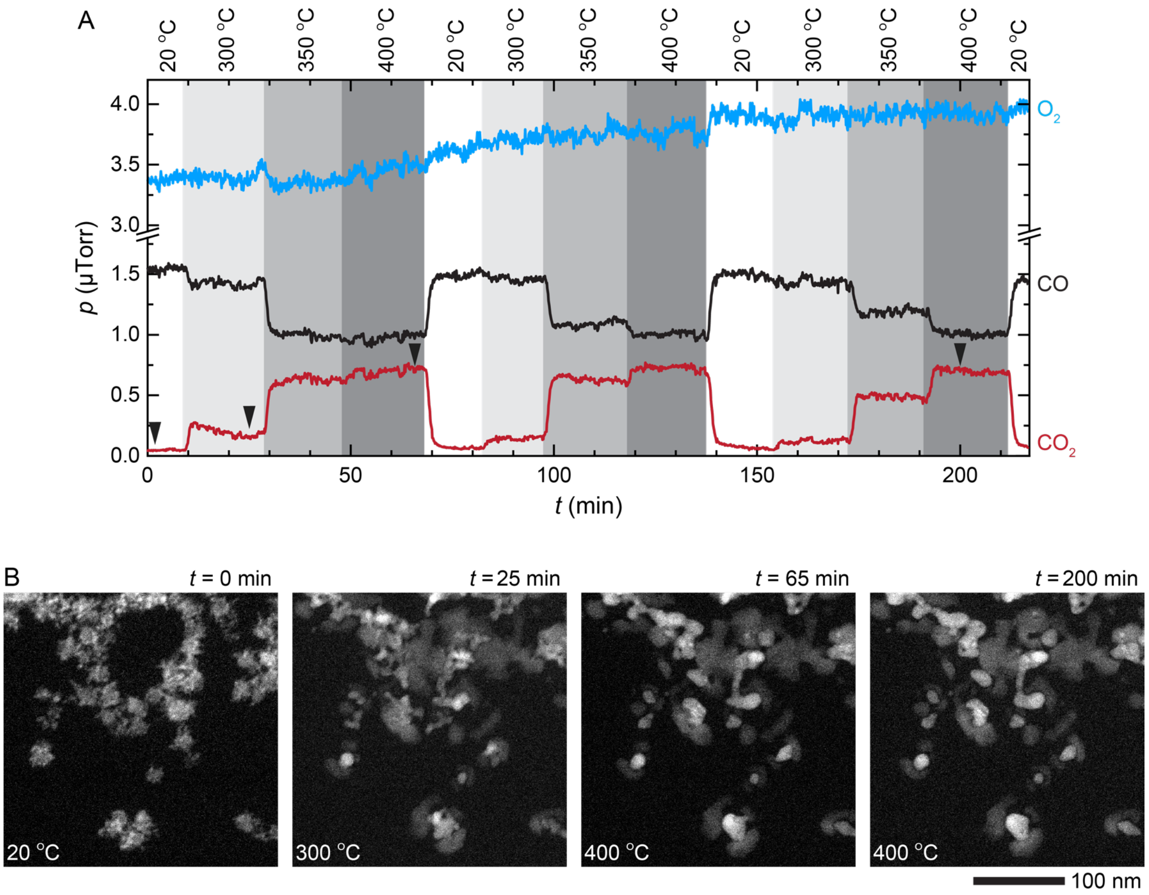

Figure S8. CO oxidation reaction over RhCu NPs in an atmosphere of $^{p_{CO}}/_{p_{O_2}} \approx 0.5$. (**A**) Changes in the gas composition during the CO oxidation reaction in an atmosphere of $^{p_{CO}}/_{p_{O_2}} \approx 0.5$ (760 Torr of 9% CO, 18% O_2, and 73% He) at 20–400 °C. The reaction was held at each temperature (*i.e.*, 300, 350, and 400 °C) for approx. 20 min. The black arrows correspond to the time points of the image series shown in (B). (**B**) *In situ* STEM image series of the NPs during the reactions described in (A).

To verify that the catalytic activities obtained from *in situ* TEM studies are relevant, we also tested the activities of RhCu and pure Rh NPs during the CO oxidation reaction using a conventional flow reactor (Figure S9). Both Rh and RhCu NPs displayed 100% CO conversion at 200 °C.

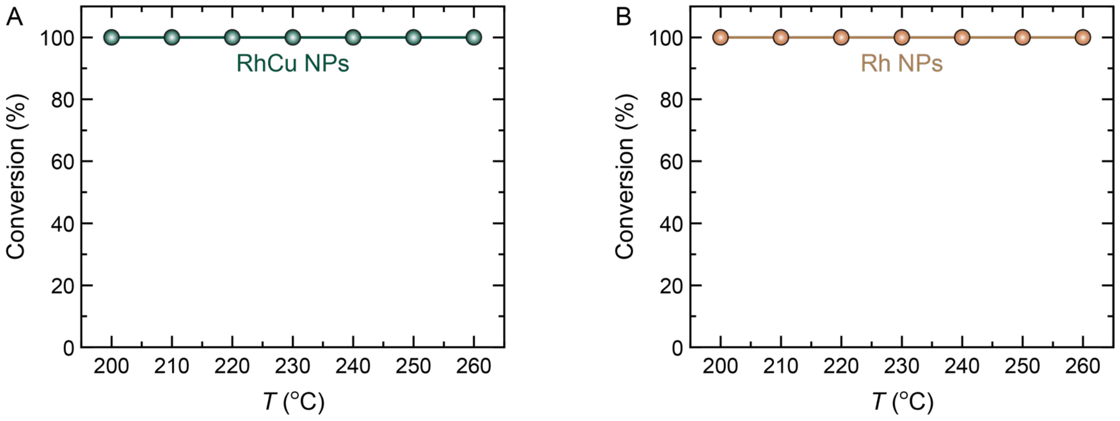

Figure S9. CO oxidation activity of the RhCu and Rh NPs. Temperature-dependent catalytic performance of (**A**) RhCu and (**B**) Rh NPs during CO oxidation reaction measured in an atmosphere of $p_{CO}/p_{O_2} \approx 0.5$ (760 Torr of 9% CO, 18% O_2, and 73% N_2) at 200–260 °C.

6. Electron diffraction images of PdCu and RhCu NPs during reactions

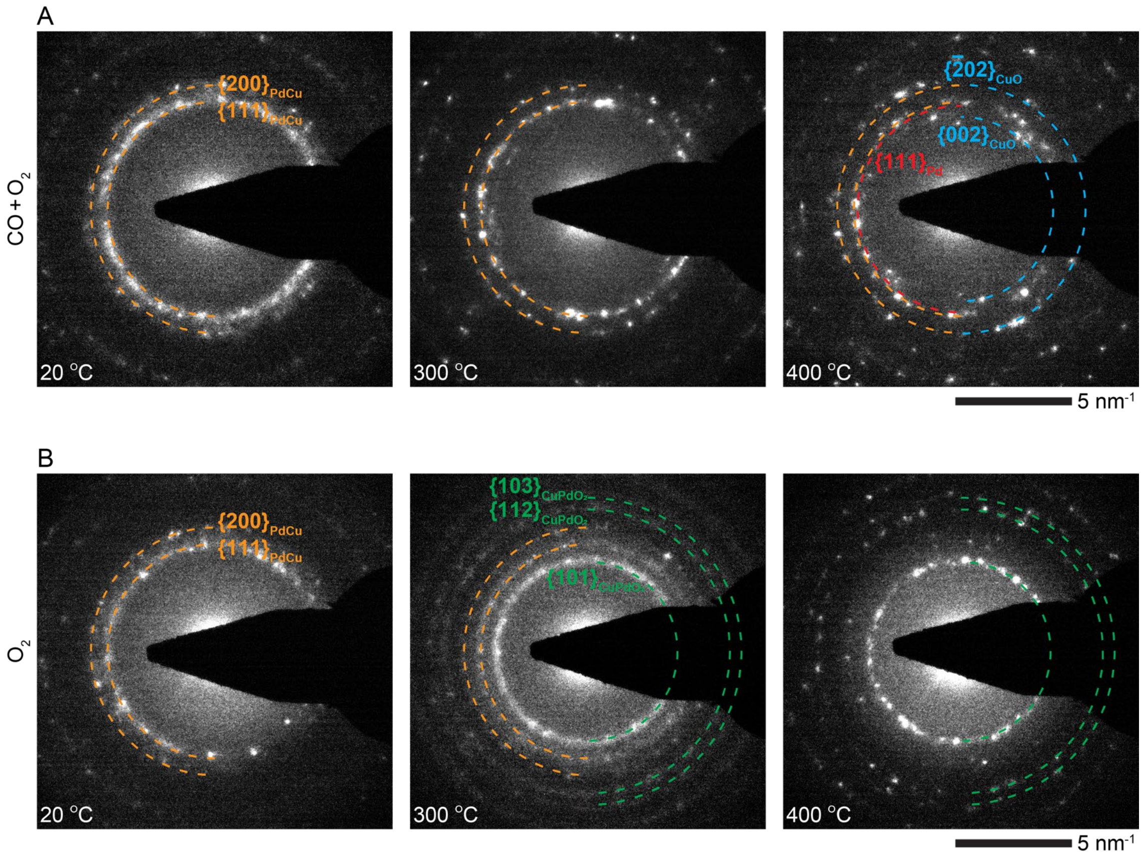

Figure S10. Electron diffraction images. Set of diffraction images corresponding to the profiles shown in (**A**) Figure 2B and (**B**) Figure 2C.

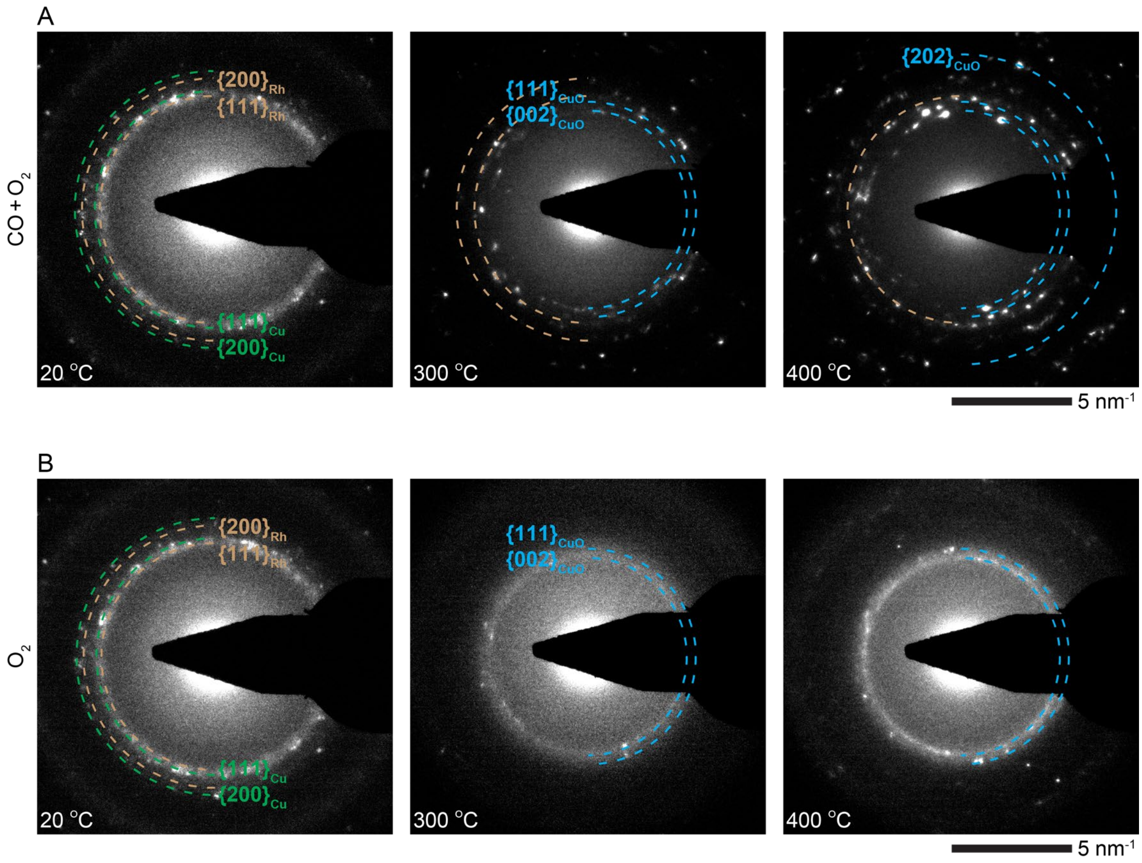

Figure S11. Electron diffraction images. Set of diffraction images corresponding to the profiles shown in (**A**) Figure S7B and (**B**) Figure S7C.

7. Supporting video captions

Supporting Video 1: Segregation of the PdCu NPs during CO oxidation reaction in an atmosphere of $^{p_{CO}}/_{p_{O_2}} \approx 0.5$ (*i.e.*, 760 Torr of 9% CO, 18% O_2, and 73% He) at 400 °C, as shown in Figure 3A.

Supporting Video 2: Oxidation of a PdCu NP in an O_2 environment (760 Torr of 20% O_2 and 80% He) at 300 °C, as shown in Figure S2A.

Supporting Video 3: Segregation of a PdCu NP during CO oxidation reaction in an atmosphere of $^{p_{CO}}/_{p_{O_2}} \approx 1.0$ (*i.e.*, 760 Torr of 17% CO, 17% O_2, and 66% He) at 400 °C, as shown in Figure S6A.

8. Supporting references

[1] A. R. Denton, N. W. Ashcroft, *Phys. Rev. A* **1991**, *43*, 3161.
[2] CrystalMaker v. 9.0.3 (CrystalMaker Software Ltd, Oxford, England). www.crystalmaker.com.
